# Adaptive Thermogenesis After Hypocaloric Low‐Carbohydrate Versus Low‐Fat Diets in African American Women: A Secondary Analysis

**DOI:** 10.1002/oby.70020

**Published:** 2025-09-10

**Authors:** Silvia Y. Lopez Torres, Barbara A. Gower, W. Timothy Garvey, Catia Martins

**Affiliations:** ^1^ Department of Nutrition Sciences University of Alabama at Birmingham (UAB) Birmingham Alabama USA

**Keywords:** energy expenditure, glycemic index, macronutrient composition, metabolic adaptation, weight loss

## Abstract

**Objective:**

This secondary analysis was conducted to compare the magnitude of adaptive thermogenesis (AT) following hypocaloric low‐carbohydrate (CHO) versus low‐fat diets in African American (AA) women.

**Methods:**

Sixty‐nine AA women with obesity were randomized to low‐CHO or low‐fat hypocaloric diets for 10 weeks, followed by a 4‐week weight stabilization period (all food provided). At baseline and Week 13, insulin sensitivity (*S*_I_) was measured by intravenous glucose tolerance test, body composition by bioimpedance analysis, total energy expenditure (EE) (TEE) by doubly labeled water, and resting EE (REE) by indirect calorimetry.

**Results:**

Forty women finished the intervention, with an average weight loss of 6.3 ± 3.9 kg and no differences between groups. AT was present at the level of REE (−75 ± 136 kcal/day, *p* < 0.003, *n* = 38), but not TEE (−80 ± 288 kcal/day, *p* = 0.086, *n* = 40) at Week 13. Women with low *S*_I_ on the low‐fat diet showed greater AT at the level of TEE compared to those on the low‐CHO diet (−202 ± 213 vs. 127 ± 239 kcal/day, respectively, *p* < 0.006).

**Conclusions:**

AA women with low *S*_I_ on low‐CHO diets do not experience AT, which can contribute to the superiority of this dietary approach in inducing weight and fat mass loss.

**Trial Registration:**

ClinicalTrials.gov Identifier: NCT03499509 https://clinicaltrials.gov/study/NCT03499509

**Study Importance**

- What is already known?
  ○ African American (AA) women are disproportionally burdened by obesity, likely due to their unique phenotype characterized by low insulin sensitivity (*S*_I_) and high acute insulin response to glucose.
  ○ Low‐carbohydrate (CHO) (vs. low‐fat) diets usually result in greater weight and fat mass (FM) loss in AA women, and differential changes in energy expenditure (EE) seem to be involved.
- What does this study add?
  ○ AA women on a hypocaloric low‐CHO diet do not experience adaptive thermogenesis (AT) at the level of total EE (TEE), while those on a low‐fat diet do.
  ○ Additionally, in AA women with low *S*_I_ at baseline randomized to a low‐CHO diet, the reduction in TEE observed with weight loss was less than predicted, while the opposite was seen in those on a low‐fat diet.
  ○ AA women with higher baseline *S*_I_ and fasting leptin and thyroid‐stimulating hormone serum concentrations experience a greater degree of AT at the level of TEE.
- How might these results change the direction of research or the focus of clinical practice?
  ○ The absence of AT at the level of TEE following weight loss in AA women on low‐CHO diets might help explain the superiority of this dietary approach in this population. This effect was especially marked in those with low *S*_I_ at baseline, where the reduction in TEE was less than predicted given the loss of FM and fat‐free mass.
  ○ These findings may help design tailored precision nutrition interventions for individuals with obesity, depending on their baseline *S*_I_, as well as help identify individuals at risk of experiencing a large AT in response to weight loss, so that strategies can be implemented to improve dietary adherence and weight loss outcomes.

## 1 Introduction

African American (AA) women are disproportionately burdened by obesity [1], with a higher prevalence compared to European Americans (55% vs. 38%) [2], which has been attributed in part to a metabolic profile characterized by lower insulin sensitivity (*S*_I_) and higher acute insulin response to glucose (AIRg) [3]. Insulin facilitates fat deposition and inhibits adipose tissue lipolysis [4, 5], while stimulating glucose uptake and promoting glycogen synthesis and glucose oxidation [6]. As such, consistently high insulin concentrations have been shown to increase adiposity and, over time, contribute to obesity [7].

Additionally, insulin plays a role in energy expenditure (EE) [8]. While insulin acutely augments thermogenesis in humans, partial knockout of the insulin gene in mice, resulting in chronic hypoinsulinemia, was associated with an increase in EE [9]. Research shows that low‐carbohydrate (CHO)/low‐glycemic diets lower insulin concentrations—resulting in a greater increase in total EE (TEE) and resting EE (REE) following weight loss maintenance compared to low‐fat diets [10], particularly in those with high post‐challenge insulin [11]. However, the relationship between baseline insulin and changes in EE in response to hypocaloric diets remains unknown.

Low‐CHO diets have gained popularity due to their ability to promote weight loss. Given the high post‐challenge insulin observed in AA women, low‐CHO diets might be beneficial in this patient group by minimizing fasting [12] and postprandial insulin secretion [13], while increasing EE. In fact, low‐CHO (vs. low‐fat) diets have been shown in several studies to result in greater weight loss in AA individuals [14, 15, 16]. Although the mechanism through which low‐CHO diets produce greater weight loss is not clear, their effect may be mediated in part by preventing or minimizing the decrease in EE associated with weight loss [10, 17, 18].

Weight loss is associated with decreases in EE that are both related to, and independent of, loss of metabolically active tissue. The term “adaptive thermogenesis” (AT) is used to describe the decrease in EE that is independent of tissue loss. AT has previously been shown to contribute to resistance to weight loss [19, 20, 21]. Whether low‐CHO diets can reduce AT during weight loss has not been investigated. Therefore, the aim of this secondary analysis was to compare AT in AA women randomized to a low‐CHO versus low‐fat diet and to determine the extent to which baseline *S*_I_ interacted with diet to determine AT.

## 2 Methods

### 2.1 Study Design

This secondary analysis included data previously collected from the parent study: A Clinical Health Approach that Motivates Participation and Inspires Others through Nutrition (CHAMPION), a two‐arm prospective randomized control trial (RCT). The main aim of this study was to investigate the effects of two hypocaloric diets, low‐CHO or low‐fat, on both weight loss and weight loss maintenance in AA women with obesity [22].

### 2.2 Participants

Adult AA women with obesity were recruited from the local communities (the University of Alabama at Birmingham [UAB], the city of Birmingham, and Jefferson and Shelby counties) between June 2019 and August 2023. Inclusion criteria included AA women (race was self‐reported); BMI 30–45 kg/m^2^; age 19–65 years; being sedentary to moderately active; and normal menstrual cycles in premenopausal women. Exclusion criteria included a history of eating disorders; daily use of tobacco (> 1 pk/week); change in weight > 5 lb in the previous 3 months; and presence of any condition or medication deemed by the study physician to interfere with research outcomes.

The study was approved by UAB IRB‐300001324, registered in ClinicalTrials.gov (NCT03499509), and conducted according to the guidelines laid down in the Declaration of Helsinki. All participants provided written informed consent before enrolling in the study.

### 2.3 Detailed Protocol

After baseline measurements, participants were randomized to either a low‐CHO (*25% CHO, 55% fat, 25% protein*) or low‐fat (*55% CHO, 20% fat, 25% protein*) diet for 10 weeks [22]. Energy requirements were estimated by multiplying REE, measured with indirect calorimetry, by 1.5 (physical activity [PA] factor), and all participants received an individualized dietary prescription throughout the weight loss phase (40% energy restriction) and during the 4‐week weight stabilization period (energy balance [EB]), with all food provided [22].

### 2.4 Measures of Adherence

Participants completed weekly food checklists, reviewed by the study dietitian. During both phases, adherence was assessed using a panel of biochemical (ketones, beta‐hydroxy‐butyrate [βHB], and triglycerides) and physiological (body weight, respiratory quotient [RQ]) measures [22]. Additionally, participants were asked to maintain their baseline level of PA throughout the study.

### 2.5 Testing

The following variables were measured at baseline and Week 13, unless otherwise stated, and assessments were performed in the Core facilities of the Center for Clinical and Translational Science (CCTS), Nutrition Obesity Research Center (NORC), and Diabetes Research Center (DRC) at UAB. Participants were asked to avoid strenuous PA the day prior to testing and all PA on the morning of testing. All insulin sensitivity measurements were performed in the Clinical Research Unit (CRU) at UAB's CCTS after an overnight fast of 12 h.

*TEE* was measured through the CCTS/NORC/DRC Core Laboratory by doubly labeled water (DLW) following established procedures [22, 23]. A food quotient of 0.85 was used at baseline to reflect a standard omnivorous diet, while at Week 14, the FQ of the intervention diets, calculated using the equation of Black et al. [24], was used.

*REE* was determined in the fasting state in the CCTS/NORC/DRC Core Laboratory by indirect calorimetry (TrueOne 2400, Parvo Medics, Sandy, UT).

*Body composition* (fat mass [FM] and fat‐free mass [FFM]) was measured via bioelectrical impedance analysis (BIA) (Seca 514 mBCA, Seca GmHb & Co. KG, Hamburg, Germany).

*S*_I_ was measured at baseline only with an intravenous glucose tolerance test (IVGTT) and minimal modeling, as previously described [25], but using 300 mg of glucose (20% dextrose)/kg of body weight. The IVGTT was performed at the CRU after a 12‐h fast. AIRg was calculated as the incremental insulin area‐under‐the‐curve from minutes 0 to 10 following glucose injection using the trapezoidal method.

*For laboratory analyses*, blood samples were collected in the fasting state, and concentrations of insulin and TSH were measured using a TOSOH A1A‐900 immunoassay analyzer (TOSOH Bioscience, South San Francisco, CA), leptin using R&D Systems (Minneapolis, MN) Human Leptin Quantikine ELISA kits, and glucagon using MesoScale Discovery (Rockville, MD) Human Metabolic Panel I kits in the CCTS/DRC Core Laboratory.

### 2.6 Statistical Analysis

Statistical analysis was performed with SPSS version 29 (SPSS Inc., Chicago, IL), and data were presented as mean ± SD in text and tables and SEM in figures unless otherwise stated. Statistical significance was set at *p* < 0.05, and normality of variables was assessed by visual inspection of the histogram and Shapiro–Wilk test. *S*_I_ and TSH concentrations were not normally distributed and were log transformed. Changes in body composition and TEE from baseline to Week 13 were analyzed with a paired samples *t*‐test performed in participants where AT at the level of TEE was greater than −1000 kcal/day. Differences between groups were evaluated with an independent samples *t*‐test in the same sample. Additionally, changes over time among participants who completed all three time points were assessed using a repeated measures general linear model (Table S1).

The presence of AT at the level of TEE and REE was tested by paired samples *t*‐test comparing measured EE (TEE_m_ and REE_m_) with predicted EE (TEE_p_ and REE_p_) at the same time points.

An equation to predict TEE was derived from all participants with TEE data at baseline (*n* = 48).

$$\begin{array}{c} \text{Model}:{\mathrm{TEE}}_{p}\;\left(\text{kcal}/\mathrm{day}\right)=10.86+\left(4.10\times \mathrm{Age}\;\left[\text{years}\right]\right)\\ \;-\left(5.43\times \mathrm{FM}\;\left[\mathrm{kg}\right]\right)\\ \;+\left(45.18\times \mathrm{FFM}\;\left[\mathrm{kg}\right]\right) \end{array}$$

*n* = 48; *R* ^2^ = 0.41; *p* < 0.001.

An additional equation to predict REE was derived from all participants with REE data at baseline (*n* = 59).

$$\begin{array}{c} \text{Model}:{\mathrm{REE}}_{p}\;\left(\text{kcal}/\mathrm{day}\right)=728.02-\left(4.79\times \mathrm{Age}\;\left[\text{years}\right]\right)\\ \;+\left(2.97\times \mathrm{FM}\;\left[\mathrm{kg}\right]\right)\\ \;+\left(15.79\times \mathrm{FFM}\;\left[\mathrm{kg}\right]\right) \end{array}$$

*n* = 59; *R* ^2^ = 0.59; *p* < 0.001.

To test the main aim of the study, ANCOVA was used. The primary analysis treated *S*_I_ as a continuous variable and examined both the independent and interactive effects of diet and *S*_I_ on AT at the level of TEE and REE. Further subgroup analyses to identify significant differences among groups were conducted with one‐way ANOVA and independent sample *t*‐tests, where *S*_I_ was categorized as “high” or “low” *S*_I_ based on the median cutoff of 1.89 (×10^−4^ min^−1^/[μIU/mL]).

To investigate if AT was associated with baseline metabolic health, bivariate correlations were conducted between AT at the level of TEE/REE and several endocrine measures (fasting insulin, leptin, glucagon, TSH, *S*_I_, AIRg, and disposition index [DI]). Prior to analysis, extreme values (≥ 3 SD from the mean) were excluded from three participants based on their fasting baseline insulin serum concentrations, one based on TSH, and one based on AIRg and DI. Correlation analyses were performed in all participants combined, as well as separately within each diet group. Additionally, linear regression analyses were conducted to determine if *S*_I_, leptin, and TSH were predictors of AT at the level of TEE and REE after adjusting for diet group and menopause status.

## 3 Results

**FIGURE 1:**
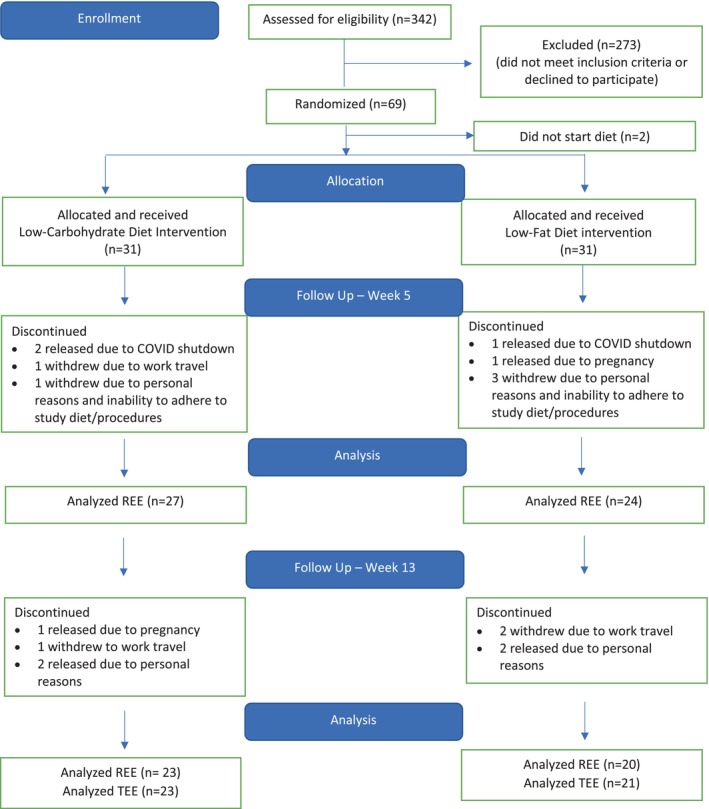
Flowchart of the study. [Color figure can be viewed at wileyonlinelibrary.com]

A CONSORT diagram of the CHAMPION study is shown in Figure 1. Forty women aged 48 ± 10 years with BMI of 38 ± 6 kg/m^2^ finished the 14‐week intervention. Overall, there was a significant reduction in body weight (6.35 ± 3.90 kg), FM (4.74 ± 3.35 kg), and FFM (1.61 ± 1.82 kg) from baseline to Week 13, with no significant differences between diet groups (Table 1). Additionally, there were no significant changes in body weight during the stabilization period (0.26 ± 1.33 kg, *p* = 0.180).

**TABLE 1:** Anthropometrics and energy expenditure at baseline and Week 13 by diet group.

	Low‐CHO diet (*n* = 22)		Low‐fat diet (*n* = 18)	
	Baseline	Week 13	Baseline	Week 13
BMI (kg/m^2^)	38.8 ± 6.6	36.7 ± 6.2***	36.5 ± 4.5	34.5 ± 4.7***
Body weight (kg)	106.1 ± 16.2	100.0 ± 14.6***	102.8 ± 16.6	96.7 ± 16.8***
FM (kg)	52.1 ± 12.2	47.6 ± 10.9***	49.4 ± 11.0	44.4 ± 10.3***
FFM (kg)	54.4 ± 5.4	52.4 ± 5.1***	53.5 ± 6.8	52.4 ± 7.9**
TEE_m_ (kcal/day)	2412 ± 350	2311 ± 369	2340 ± 291	2158 ± 396**
TEE_p_ (kcal/day)	2373 ± 186	2307 ± 173**	2365 ± 249	2342 ± 297
AT TEE (kcal/day)	39 ± 274	5 ± 294	−24 ± 226	−183 ± 252^##^

*Note*: Data presented as mean ± SD. Asterisk represents significant changes over time within each group (paired samples *t*‐tests): *p < 0.05, **p < 0.01, ***p < 0.001. The changes in anthropometrics and EE over time were not statistically different between groups (independent samples *t*‐test). Differences between measured and predicted EE were calculated with paired samples *t*‐tests: ^##^ *p* < 0.01.

Abbreviations: AT, adaptive thermogenesis; CHO, carbohydrate; FFM, fat‐free mass; FM, fat mass; TEE, total energy expenditure; TEE_m_, measured TEE; TEE_p_, predicted TEE.

Overall, there was a trend for TEE_m_ to be lower than TEE_p_ at Week 13 (−80 ± 288 kcal/day, *p* = 0.086, *n* = 40). AT at the level of REE at Weeks 5 and 13 was statistically significant, with REE_m_ being significantly lower than REE_p_ (−61 ± 115 kcal/day, *p* < 0.001, *n* = 49 and −75 ± 136 kcal/day, *p* = 0.002, *n* = 38, respectively).

**FIGURE 2:**
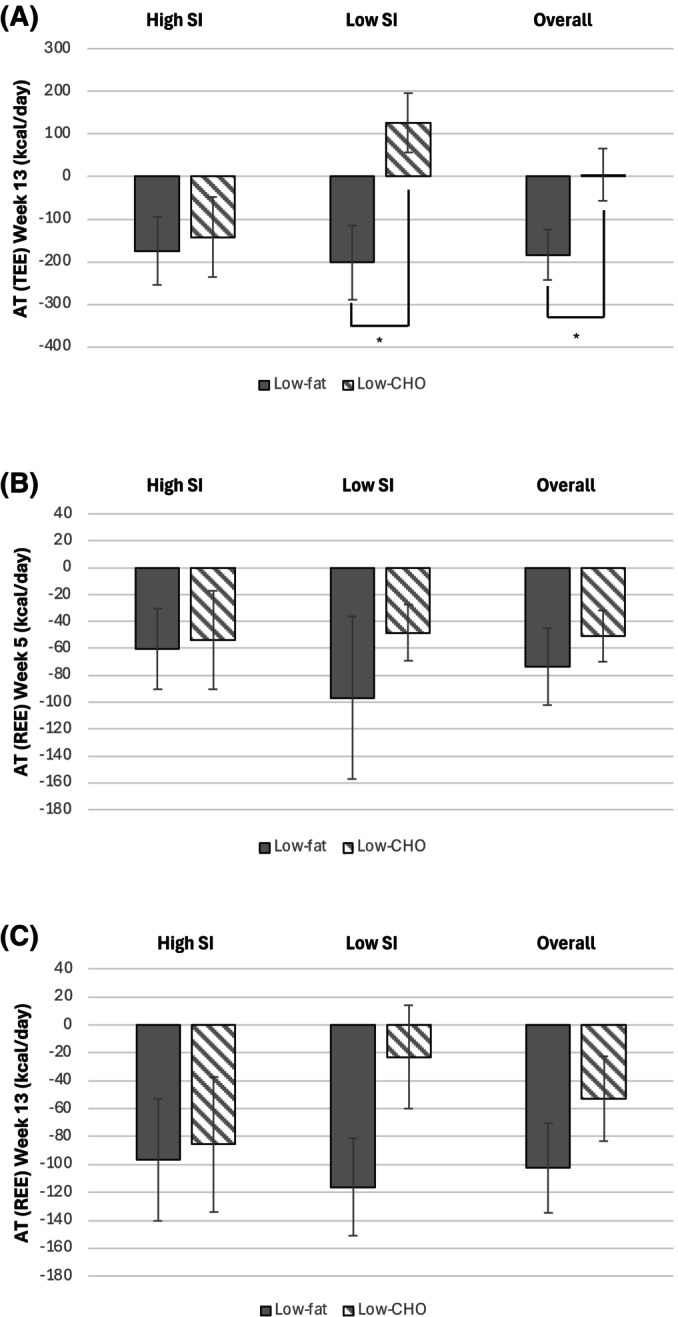
Adaptive thermogenesis (AT) at the level of resting energy expenditure (REE) at (B) Week 5 and (C) Week 13 and (A) total energy expenditure (TEE) at Week 13 (C) in African American women with high or low insulin sensitivity (*S*_I_) at baseline randomized to a low‐fat or low‐carbohydrate (CHO) diet. *Denotes significant differences between groups (*p* < 0.05). The primary analyses treated *S*_I_ as a continuous variable to determine if there was an effect of diet, *S*_I_, or their interaction on AT at the level of TEE or REE in an ANCOVA model. For subgroup analyses, the median *S*_I_ 1.89 (×10^−4^ min^−1^/[μIU/mL]) was used to designate status, that is, high versus low *S*_I_. For graphical representation, differences in AT at the level of TEE and REE among *S*_I_ and diet groups were determined using ANOVA. Data presented as mean ± SE.

Figure 2 displays AT at the level of TEE and REE in women with high versus low *S*_I_ randomized to low‐fat versus low‐CHO diets. A significant diet effect (*p* = 0.003) and a diet*S_I_ interaction (*p* = 0.017) were seen on AT at the level of TEE at Week 13. However, no statistically significant effect of *S*_I_ was observed on AT (*p* = 0.687). Moreover, subgroup analysis showed that women with low *S*_I_ randomized to the low‐fat diet experienced greater AT at the level of TEE (−202 ± 213 kcal/day) compared to those with low *S*_I_ on the low‐CHO diet (127 ± 239 kcal/day, *p* = 0.006), and that overall, those on a low‐CHO diet experienced less AT compared to those on the low‐fat diet (5 ± 294 kcal/day vs. −184 ± 252 kcal/day, *p* = 0.019) (Figure 2A). Using RQ instead of FQ in our analysis did not change the results (data not shown). A scatterplot for the association between AT at the level of TEE at Week 13 (kcal/day) and baseline *S*_I_ by diet group is shown in Figure S1.

No significant effect of diet (*p* = 0.817 and *p* = 0.340, respectively), *S*_I_ (*p* = 0.351 and *p* = 0.646, respectively), or their interaction (*p* = 0.865 and *p* = 0.548, respectively) was seen on AT at the level of REE, either at Weeks 5 or 13 (Figure 2B,C). Even though visually, AT at the level of REE appears to be greater in those with low *S*_I_ on the low‐fat compared with the low‐CHO diet, both at Weeks 5 and 13, this did not reach statistical significance (*p* = 0.347 and *p* = 0.216, respectively), likely due to the large variability in response (large SD).

**FIGURE 3:**
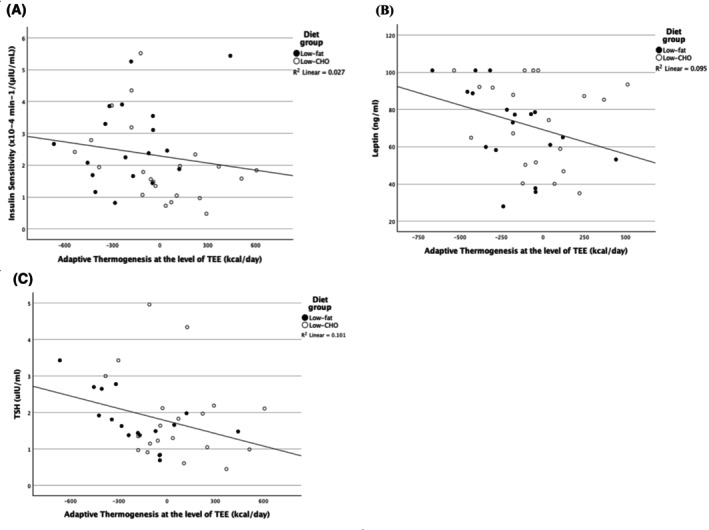
Scatterplots for the association between adaptive thermogenesis (AT) at the level of total energy expenditure (TEE) and (A) baseline insulin sensitivity (*S*_I_), (B) fasting leptin, and (C) fasting thyroid‐stimulating hormone (TSH) serum concentrations. Low‐fat diet (black filled circle) and low‐carbohydrate (CHO) diet (black open circle).

Overall, a significant inverse correlation was seen between AT at the level of TEE and baseline *S*_I_ (*r* = −0.314, *p* = 0.048, *n* = 40, Figure 3A) and fasting serum leptin concentration (*r* = −0.370, *p* = 0.022, *n* = 38, Figure 3B). However, no association between AT at the level of TEE and baseline *S*_I_ was seen in the low‐fat group, despite the association with leptin remaining significant (*r* = −0.611, *p* = 0.007, *n* = 18). The opposite was seen in the low‐CHO diet group, where the association between AT at the level of TEE and baseline *S*_I_ remained significant (*r* = −0.512, *p* = 0.015, *n* = 22), while the association with fasting leptin disappeared. Additionally, overall, there was a significant inverse correlation between AT at the level of REE at Week 13 and fasting leptin and AIRg (*r* = −0.368, *p* = 0.027, *n* = 36 and *r* = −0.491, *p* = 0.002, *n* = 38, respectively). When the diet groups were analyzed separately, no association was found between AT at the level of REE (Week 13) and fasting serum leptin concentration; however, the association remained for AIRg in the low‐CHO diet (*r* = −0.631, *p* = 0.002, *n* = 21) but disappeared in the low‐fat group. A positive association between TSH and AT at the level of TEE was observed among all subjects, despite not reaching statistical significance (*r* = −0.299, *p* = 0.072, *n* = 37, Figure 3C). Additionally, in the low‐fat diet group, the association between AT at the level of TEE and baseline TSH was significant (*r* = −0.601, *p* = 0.011, *n* = 17), but not in the low‐CHO group. At Week 13, AT at the level of TEE and REE were inversely associated with DI (*r* = −0.371, *p* = 0.020, *n* = 39 and *r* = −0.498, *p* = 0.002, *n* = 37, respectively).

Menopausal status was a significant predictor of AT at the level of TEE, after adjusting for diet group, with AT being lower in premenopausal versus postmenopausal women (42 ± 284 vs. −215 ± 232 kcal/day, respectively, *p* = 0.002). The previously reported associations between AT at the level of TEE and baseline *S*_I_, leptin, and TSH disappeared after adjusting for diet group and menopause status (Table 2, Model A–C). Overall, model C, containing TSH, diet group, and menopause status, explained 36% of the variance in AT at the level of TEE. However, when baseline *S*_I_, leptin, and TSH were all included in the model, adjusting for diet group and menopause status, only TSH was a significant predictor of AT at the level of TEE (Table 2, Model D).

**TABLE 2:** Prediction models to estimate adaptive thermogenesis at the level of TEE.

AT–TEE	Predictors	*β* coefficient (95% CI)	*p*	*R* ^2^ adjusted
Model A			0.007	0.225
	Constant	(−483.22 to 189.96)	0.383	
	*S*_I_	−0.107 (−467.76 to 222.75)	0.476	
	Diet group	0.240 (−34.98 to 308.98)	0.115	
	Menopause status	−0.401 (−394.04 to −62.89)	0.008	
Model B			0.010	0.219
	Constant	(−360.47 to 327.59)	0.923	
	Leptin	−0.253 (−6.53 to 0.61)	0.101	
	Diet group	0.258 (−20.94 to 291.87)	0.087	
	Menopause status	−0.324 (−329.83 to −9.79)	0.038	
Model C			0.001	0.364
	Constant	(−481.96 to 56.65)	0.118	
	TSH	−0.261 (−649.17 to 19.42)	0.064	
	Diet group	0.366 (52.05 to 356.54)	0.010	
	Menopause status	−0.363 (−358.74 to −46.42)	0.013	
Model D			0.003	0.349
	Constant	(−422.00 to 339.65)	0.827	
	*S*_I_	−0.074 (−400.79 to 243.41)	0.621	
	Leptin	−0.157 (−5.26 to 1.72)	0.309	
	TSH	−0.323 (−684.12 to −16.17)	0.041	
	Diet group	0.319 (11.21 to 314.05)	0.036	
	Menopause status	−0.281 (−295.84 to 8.69)	0.064	

*Note*: TSH fasting plasma concentrations were log transformed. Diet group: 1 for low‐fat and 2 for low‐CHO. Menopause status: 0 for premenopausal and 1 for postmenopausal.

Abbreviations: AT, adaptive thermogenesis; *S*_I_, insulin sensitivity; TEE, total energy expenditure; TSH, thyroid‐stimulating hormone.

## 4 Discussion

The aim of this secondary analysis was to investigate the presence, or absence, of AT at the level of TEE and REE in AA women with obesity undergoing a weight loss intervention with either a low‐CHO or low‐fat diet and to determine if diet or *S*_I_ modulated AT. AT was seen both at the level of TEE and REE, with measured EE being lower than predicted. Additionally, an interaction between diet and *S*_I_ was observed: AA women with low baseline *S*_I_ randomized to the low‐CHO diet did not experience AT at the level of REE at Week 13, and strikingly TEE at Week 13 was greater than predicted in this group. Finally, *S*_I_ and fasting leptin concentrations at baseline were associated with AT at the level of TEE.

In this study, we observed a significant diet effect and a diet*S_I_ interaction on AT at the level of TEE, with AA women with low *S*_I_ randomized to the low‐fat diet experiencing a greater AT compared to those on the low‐CHO diet. To our knowledge, this is the first study examining a potential diet*S_I_ interaction on AT. Our research group has previously shown that AA women with low *S*_I_ at baseline randomized to a low‐CHO diet lose more weight and FM compared to those with high *S*_I_ randomized to a low‐fat diet, potentially due to a smaller reduction in both TEE and REE [22]. This study adds to our previous findings by showing that the reduction in TEE and REE observed in AA women with low *S*_I_ randomized to the low‐CHO diet was not only smaller than that observed in other groups, but also smaller than expected given the greater weight and FM loss observed in that group.

Our findings demonstrate that all groups experienced a certain degree of AT at the level of REE, except for women with low *S*_I_ randomized to the low‐CHO diet. Additionally, women on the low‐CHO diet did not experience AT at the level of TEE in response to weight loss, and importantly, those with low *S*_I_ on the low‐CHO diet had a measured TEE that was greater than predicted—opposite to typical weight loss responses. These findings indicate that the differences in AT at the level of TEE might have been driven by differences in both REE and non‐REE. The non‐resting component of TEE is heterogeneous and includes diet‐induced thermogenesis (DIT) [26], non‐exercise activity thermogenesis [26], and PA‐related EE [26]. Given that the diets were matched for protein—and that protein has the highest thermic effect [27]—DIT likely did not contribute to the differences in AT between the diets. Participants were asked not to change their PA levels throughout the study, but this was not measured objectively. Additionally, participants could have become more energy efficient. Even though our research group did not find any effect of diet on mechanical efficiency using a submaximal cycling test [22], this exercise test may not be the most appropriate or reflective of normal daily life in this population. Overall, the mechanisms by which the macronutrient composition of a diet influences AT remain unknown.

Few studies have reported AT in response to diets with different macronutrient composition. In the POUNDS LOST study, AT at the level of REE was present at 6 months in participants assigned to low‐fat diets, while those on high‐fat diets experienced a small, nonsignificant AT [28]. This aligns with our results, which showed attenuated AT at the level of REE in those randomized to the low‐CHO, high‐fat diet. The POUNDS LOST study included both men and women, and 16% of the participants were AA [29]. This strengthens the generalizability of our study findings. However, TEE was not measured in POUNDS LOST, and there was low dietary adherence. In the PREVIEW study, in individuals with prediabetes, Drummen et al. investigated whether an increased protein/CHO ratio would reduce AT during weight loss maintenance. Participants underwent an initial 8‐week weight loss phase, followed by a 34‐month weight maintenance period with either a moderate‐protein or high‐protein diet, combined with moderate‐ or high‐intensity PA [30]. The high‐protein diet (25% protein, 45% CHO, 30% fat) counteracted the average AT of 120 kcal/day seen at ~34 months in the moderate‐protein (15% protein, 55% CHO, 30% fat) group [30]. This, again, is in line with our findings where a lower CHO intake was associated with less AT in response to weight loss. Despite the presence of PA in the PREVIEW study, unlike ours, PA is unlikely to have influenced the outcomes, as we have previously shown that exercise‐induced weight loss is also associated with AT [31]. Overall, the available evidence seems to suggest that lower CHO diets are associated with less AT in response to weight loss in different populations.

Several studies have examined how the macronutrient composition of the diet affects EE, both during weight loss and weight loss maintenance. Hall et al. reported no differences in EE between low‐CHO and low‐fat diets in a meta‐analysis of 32 isocaloric controlled‐feeding studies, under weight maintenance [32]. However, in a reanalysis of those studies accounting for the duration of intervention, Ludwig et al. found that on long‐term trials (> 4 weeks), TEE was increased by 135 kcal/day on low‐CHO versus low‐fat diets [17]. Low‐CHO diets have been shown to increase both TEE and REE during weight maintenance [10, 18], and despite the mechanisms, EE has not been clearly understood; ketosis might play a role. The increase in EE could be driven by increased hepatic oxygen consumption, which is proportional to the rate of ketogenesis, raising the body's needs for energy to perform gluconeogenesis and triglyceride‐fatty acid recycling [33, 34]. While not all participants were in nutrition‐induced ketosis (βHB > 0.3 mM), average βHB serum concentrations were significantly higher in the low‐CHO versus low‐fat diet group [22]. Insulin might also be involved, as a decrease in insulin secretion has been described to increase EE [9]. Low‐CHO diets have the potential to decrease insulin concentrations more than low‐fat diets, which could then lead to a greater increase, or smaller reduction, in EE in response to these diets [15]. Inconsistencies among studies might result from methodological differences in the measurement of TEE (indirect calorimetry vs. DLW), as well as the use of FQ versus RQ in the studies using DLW [35]. Despite this, the overall evidence seems to suggest that low‐CHO diets of more than 4 weeks duration increase EE under weight‐maintaining conditions or minimize the reduction observed during weight loss.

AT at the level of TEE, but not REE, was inversely associated with baseline *S*_I_ and fasting leptin and TSH in the present study. Overall, AA women with higher *S*_I_ at baseline and greater fasting leptin and TSH concentrations experienced a greater magnitude of AT at the level of TEE. Leptin modulates not only appetite but also EE [36], and a decrease in its concentrations in response to weight loss has been found to be a major determinant of AT at the level of both REE [37, 38] and TEE [39]. However, in the Biggest Loser competition, changes in leptin were not associated with AT at the level of REE [40, 41], and baseline serum leptin concentrations have similarly shown no association with EE [42], raising questions about the role of leptin in AT in humans. Moreover, leptin concentrations seem to change in response to diets varying in macronutrient composition, even in the absence of corresponding changes in FM [43]. Despite the association between *S*_I_, fasting leptin, and TSH and AT at the level of TEE in the present study, after accounting for diet group and menopausal status, the only independent predictor of AT at the level of TEE was TSH. This suggests a potential role for the thyroid axis—a regulatory feedback system involving hormones such as TSH, triiodothyronine, and thyroxine—on AT [40]. Our findings also suggest that menopausal status is a significant predictor of AT at the level of TEE at Week 13. Even though menopausal transition is associated with a decrease in EE and whole‐body fat oxidation [44], the mechanisms responsible for the greater AT in postmenopausal women remain to be determined. Overall, it seems that AT is modulated by several metabolic and hormonal markers.

The present study has several strengths. First, all food was provided during the 14 weeks of the study. Second, *S*_I_ and EE were measured with gold standard methods: IVGTT for *S*_I_, DLW for TEE, and indirect calorimetry for REE. Additionally, EE was measured after a period of weight stabilization, with participants in EB. This is important, as we have previously shown that the degree of AT is modulated by the EB status of the participants [45]. However, there are also some limitations to consider. Firstly, body composition was assessed with BIA, a two‐compartment model, which does not provide information regarding changes in FFM compartments such as skeletal muscle and organ mass. Secondly, this study was not powered to look at differences in AT between diet groups, or for a diet*S_I_ interaction. Since this is a secondary analysis, we need to be careful in the interpretation of the results, and more studies are needed to confirm these findings. Lastly, the study was restricted to AA women with obesity, which may limit the generalizability of our findings. However, previous studies in other populations [28, 30] suggest that the results might be generalizable.

## 5 Conclusion

AA women with low baseline *S*_I_ randomized to a low‐CHO diet experience less AT in response to weight loss. This offers an additional explanation for the high efficacy of these diets in inducing weight and FM loss in this population. Baseline serum concentrations of TSH may serve as a metabolic predictor of AT following weight loss. These research findings offer opportunities for tailored precision nutrition.

## Conflicts of Interest

The authors declare no conflicts of interest.

## Supporting information


**Figure S1:** Scatterplot for the association between adaptive thermogenesis (AT) at the level of total energy expenditure (TEE) at Week 13 (kcal/day) and baseline insulin sensitivity (*S*_I_) by diet group. Linear regression lines are shown for each diet group. The line for the low‐fat diet group (solid black line) had an *R* ^2^ of 0.130 and a *p* value of 0.071. The line for the low‐CHO group (dashed black line) had an *R* ^2^ of 0.168 and a *p* value of 0.029.

**Table S1:** oby70020‐sup‐0002‐TableS1.

## Data Availability

The data that support the findings of this study are available from the corresponding author upon reasonable request.
