# Supplementary figures and images for: Adaptive Thermogenesis After Hypocaloric Low‐Carbohydrate Versus Low‐Fat Diets in African American Women: A Secondary Analysis
Source: Obesity (Silver Spring). 2025 Sep 10;33(11):2160–9. doi: 10.1002/oby.70020 (PMC12559781; doi:10.1002/oby.70020)

Figure S1.

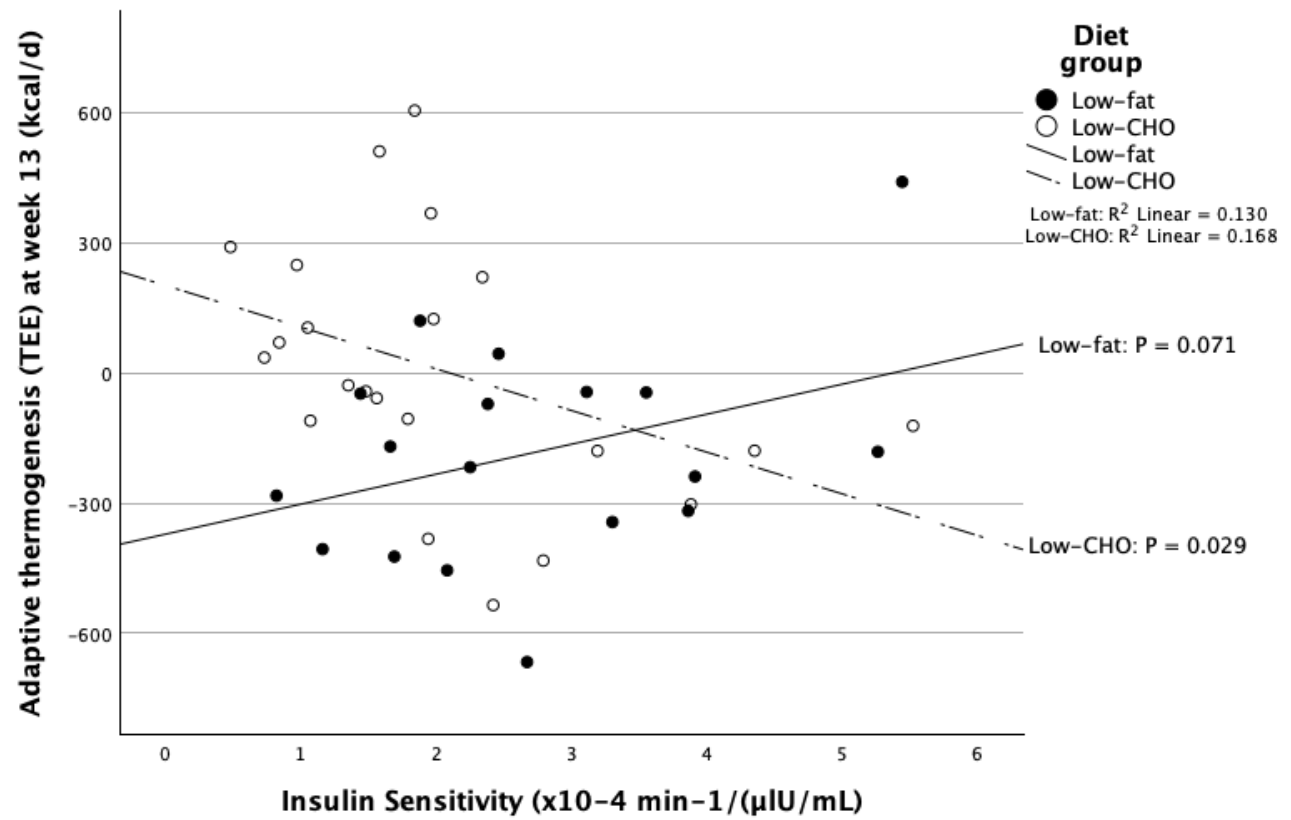

Supplement: Supplementary file 1 — Figure S1: Scatterplot for the association between adaptive thermogenesis (AT) at the level of total energy expenditure (TEE) at Week 13 (kcal/day) and baseline insulin sensitivity (S I) by diet group. Linear regression lines are shown for each diet group. The line for the low‐fat diet group (solid black line) had an R 2 of 0.130 and a p value of 0.071. The line for the low‐CHO group (dashed black line) had an R 2 of 0.168 and a p value of 0.029. [file OBY-33-2160-s001.pdf]
