# Supplementary material for: Adaptive Thermogenesis After Hypocaloric Low‐Carbohydrate Versus Low‐Fat Diets in African American Women: A Secondary Analysis
Source: Obesity (Silver Spring). 2025 Sep 10;33(11):2160–9. doi: 10.1002/oby.70020 (PMC12559781; doi:10.1002/oby.70020)
Supplement: Supplementary file 2 — Table S1: oby70020‐sup‐0002‐TableS1. [file OBY-33-2160-s002.docx]

| **Table S1.** Anthropometrics and Energy Expenditure at baseline, week 5, and week 13 by diet group | | | | | | | | | |
| --- | --- | --- | --- | --- | --- | --- | --- | --- | --- |
|  | Low-CHO Diet | | |  | Low-Fat Diet | | |  | |
|  | Baseline | Week 5 | Week 13 |  | Baseline | Week 5 | Week 13 | P-value time | P-value group |
| BMI (kg/m^2^) | 38.7±6.4 | 37.7±6.6 | 36.7±6.1 | BMI (kg/m2) | 37.2±5.4 | 36.1±5.7 | 35.3±5.9 | <0.001 | NS |
| n = 23 |  |  |  | n = 19 |  |  |  |  |  |
| Body weight (kg) | 106.2±15.7 | 102.6±14.7 | 99.8±13.9 | Body weight (kg) | 102.5±17.8 | 99.3±18.2 | 97.1±18.5 | <0.001 | NS |
| n = 25 |  |  |  | n = 21 |  |  |  |  |  |
| FM (kg) | 51.6±12.2 | 49.4±11.6 | 47.2±10.8 | FM (kg) | 48.7±11.0 | 46.4±10.1 | 44.3±10.0 | <0.001 | NS |
| n = 23 |  |  |  | n = 18 |  |  |  |  |  |
| FFM (kg) | 54.4±5.3 | 53.0±5.1 | 52.5±5.0 | FFM (kg) | 53.2±6.3 | 52.2±6.5 | 52.0±7.0 | <0.001 | NS |
| n = 23 |  |  |  | n = 18 |  |  |  |  |  |
| REE_m_ (kcal/d) | 1541±216 | 1454±179 | 1442±236 | REE_m_ (kcal/d) | 1492±133 | 1394±166 | 1359±143 | <0.001 | NS |
| n = 22 |  |  |  | n = 18 |  |  |  |  |  |
| REE_p_ (kcal/d) | 1521±146 | 1493±141 | 1477±139 | REE_p_ (kcal/d) | 1467±130 | 1444±135 | 1434±146 | <0.001 | NS |
| n = 23 |  |  |  | n = 18 |  |  |  |  |  |
| Data presented as mean $\pm$ SD. N: sample size; CHO: carbohydrate: BMI: body mass index; FM: fat mass; FFM: fat-free mass; REE_m_: measured REE; REE_p_: predicted REE. There were significant differences among all variables over time (P < 0.001), with no significant time*group interaction. Table includes participants who completed all three timepoints. | | | | | | | | | |
